# Evaluation of Fluid Behaviors in a Pushbutton-Activated Microfluidic Device for User-Independent Flow Control

**DOI:** 10.3390/mi15040465

**Published:** 2024-03-29

**Authors:** Dong Hyun Han, Gihyun Lee, Untaek Oh, Yejin Choi, Je-Kyun Park

**Affiliations:** 1 Department of Bio and Brain Engineering, Korea Advanced Institute of Science and Technology (KAIST), 291 Daehak-ro, Yuseong-gu, Daejeon 34141, Republic of Korea; honna94@kaist.ac.kr (D.H.H.); gihyunlee@kaist.ac.kr (G.L.); untaek624@kaist.ac.kr (U.O.); yejin9343@kaist.ac.kr (Y.C.); 2 KI for Health Science and Technology, KAIST Institutes (KI), 291 Daehak-ro, Yuseong-gu, Daejeon 34141, Republic of Korea; 3 KI for NanoCentury, KAIST Institutes (KI), 291 Daehak-ro, Yuseong-gu, Daejeon 34141, Republic of Korea

**Keywords:** pushbutton-activated microfluidic device, point-of-care testing, power-free microfluidics, flow behavior

## Abstract

Although numerous studies have been conducted to realize ideal point-of-care testing (POCT), the development of a user-friendly and user-independent power-free microfluidic platform is still a challenge. Among various methods, the finger-actuation method shows a promising technique that provides a user-friendly and equipment-free way of delivering fluid in a designated manner. However, the design criteria and elaborate evaluation of the fluid behavior of a pushbutton-activated microfluidic device (PAMD) remain a critical bottleneck to be widely adopted in various applications. In this study, we have evaluated the fluid behavior of the PAMD based on various parameters, such as pressing velocity and depth assisted by a press machine. We have further developed a user-friendly and portable pressing block that reduces user variation in fluid behavior based on the evaluation.

## 1. Introduction

Microfluidic technology is one of the key technologies widely studied to realize ideal point-of-care testing (POCT) due to various advantages such as the small reagent volume consumption, the integration of multiple functions into a single device, and efficient reaction [1,2,3,4]. Advances in the microfluidic POCT have the potential to overcome the limitations of the traditional diagnostic technologies suffering from bulky equipment, lengthy sample preparation, and the requirement for trained personnel [5,6,7], as well as the simple and cost-effective approaches exhibiting a relatively high false negative ratio of reaction [8,9,10]. In addition, the microfluidic POCT technology satisfies the acronym “ASSURED”, previously suggested by the World Health Organization, which stands for affordable, sensitive, specific, user friendly, rapid and robust, equipment free, and deliverable to end-users. The ASSURED concept has recently been updated to “RE-ASSURED” by adding real-time connectivity and ease of specimen collection to meet the increasing demand for on-site diagnostic testing [11,12,13].

Despite the remarkable development of microfluidic diagnostic technologies, the requirement of the external energy and pumping system in microfluidic technologies hinders the user-friendliness of the diagnostic tool. In response, various power-free microfluidic platforms have been reported utilizing various principles, including vacuum pumps [14,15,16], capillary channels [17,18,19], gas-driven flow [20,21,22], and pushbutton activation [23,24,25], to deliver fluid in a designated manner. Among the various methods, the pushbutton activation method provides a great leap of advancement in user-friendliness that can be operated by simply pushing and releasing a button in various environments without external equipment [26,27,28]. A pushbutton-activated microfluidic device (PAMD) uses the indirect pressurization of the poly(dimethylsiloxane) (PDMS) membrane between the fluidic channel and pneumatic channel to supply fluid samples to the microchannels controlling the dispensed volume. The developed PAMD was applied in various sample preparation applications, such as plasma separation and reaction for the blood cross-matching test [26], nucleic acid extraction [29], and droplet generation [30]. However, one of the biggest challenges of PAMD is to reduce user-dependent variation in flow behavior, which may significantly affect the performance of sample preparation [31,32,33]. Although flow behavior can be passively regulated by adjusting the channel geometry, the user-dependent variation of the flow profile remains a critical bottleneck of the PAMD to be widely adopted. Beyond simply transporting fluid in a designated manner in the PAMD, a design strategy that enables elaborate control of fluidic motion in the PAMD is required for wide applications [34]. In addition, a sophisticated operational evaluation of the PAMD is lacking.

In this paper, we performed a mechanical test of the PAMD to evaluate the flow profiles under various parameters. These parameters include design parameters of the PAMD such as channel and pushbutton dimensions, as well as human parameters such as pressing velocity, diameter, and depth. To represent the variation of human finger press, we used a customized pressing machine that could programmably push and release a button of the PAMD with precision. With the intricate control of the parameters, we could characterize several parameters, such as pressing velocity and depth, significantly affecting the flow behavior of the PAMD. Furthermore, based on the mechanical evaluation via the customized pressing machine, we developed a user-friendly PAMD that can sophisticatedly control the flow behaviors and reduce user-dependent variation by using a pressing block on the pushbutton. We believe that this study provides a fundamental understanding of the working mechanism and design criteria of PAMD and represents a cornerstone of power-free microfluidics that enables precise control without the aid of bulky and expensive equipment.

## 2. Materials and Methods

### 2.1. Silicon Wafer Micropatterning

The double-polished p-type wafer was cleaned prior to micropatterning. The SU-8 2100 (K1 solution, Kwangmyung, Republic of Korea) photoresistor was spin-coated on the wafer at 1500 rpm for 30 s (target thickness = 100 μm). The SU-8 spin-coated wafer was incubated at 65 °C for 5 min and 90 °C for 20 min. An MA6 aligner (Karl-Süss, Garching, Germany) exposed an ultraviolet (UV) light with 16 mW for 10 s. The wafer was further incubated at 65 °C for 5 min and 90 °C for 12 min. The micropatterned wafer was immersed in an SU-8 developer for 10 min to remove the unexposed photoresistor.

### 2.2. Fabrication of a Pushbutton-Activated Microfluidic Device (PAMD)

PDMS (Dowhitech, Goyang, Republic of Korea) and its curing agent (Dowhitech) were mixed in a ratio of 10:1. The mixed PDMS was poured into the micropatterned Si wafer, and the PDMS-poured wafer was incubated in an oven at 85 °C for 40 min. For the PDMS membrane, PDMS and curing agent were mixed at a 7:1 ratio, spin coated at 1500 rpm for 1 min, and baked at 120 °C for 1 min. After each layer was prepared, oxygen plasma was treated for 1 min at each layer, and the layers were permanently bonded via direct contact. After the bonding, the fabricated microfluidic device was incubated in an oven at 65 °C for 10 min for bonding stabilization. The default length of the microchannel (L) was designed as 13.5 mm, and it was measured from V2 to outlet. The lengths of the increased microchannel of PAMD were also measured in the same way. Before use, the button of the microfluidic device was pushed, and the air outlet was blocked by an air plug.

### 2.3. 3D Printing of a Press Rod and a Pressing Block

A press rod was made to apply the pressure instead of a human finger. The diameter of the pressing part of the press rod was set as 5, 7, and 10 mm to mimic a human finger size. A pressing block was made to aid a user in controlling the depth of a pushbutton. The diameter and thickness of the main body were set as 12 mm and 1 mm, respectively, and the depth of the protruding part was set as 0.4, 0.6, 0.8, 1.0, 1.2, 1.4, 1.6, and 2.0 mm. A 3D structure of the press rod and pressing block was designed using Autodesk Inventor (Autodesk, San Francisco, CA, USA). The design file was sent to a DLP 3D printer (Pro 4K65; Asiga, Alexandria, Australia), which fabricated the desired 3D structure using a PlasCLEAR resin (Asiga). The slice thickness was set as 50 μm, and the UV light was irradiated at 380 nm to cure the resin. After printing was completed, the printed 3D structure was rinsed in 100% isopropanol for 10 min and exposed to UV light for 30 min in a flash UV chamber (Asiga).

### 2.4. Customized Pressing Machine

To repeatedly and precisely control the press and release process of the button of the PAMD, the pressing machine was prepared by changing the head of XYZ gantry (KP3S, Kingroon Tech Co., Shenzhen, China) with the press rod, which can replace the human finger, referring to the previous protocol for customizing the gantry [35]. For press and release control of the machine, G-Code was utilized to set the coordinates considering the pressing depth and moving speed of the press rod. To match the location of the press rod with the PAMD button, we calibrated the machine prior to use. The press machine was operated according to the programmed command to adjust the depth and speed, which are the key parameters when pushing the button with a human finger. The reproducibility and stability of the developed press machine were validated by comparing the fluid behavior to the human finger group when pressing and releasing the button repeatedly for 80 s. After the validation of the press machine, the key parameters, including depth, speed, and rod diameter, were changed to investigate the relation with fluid behaviors that occurred in the fluid channel of the PAMD. For all the experiments, the pressing depth was set at 2.0 mm, unless otherwise specified.

### 2.5. Flow Rate and Pressure Measurements

The outlet of the PAMD was connected to a tube (outer diameter = 1.7 mm, inner diameter = 0.7 mm), and the tube was connected to a flow rate sensor (Fluigent, Jena, Germany) that measures the flow rate up to 1000 μL/h or a pressure sensor (Elveflow, Paris, France) that measures the pressure up to 1 psi or approximately 69 mbar depending on the use. The data acquisition time interval was set as 0.1 s. Distilled water was continuously pushed into and released from the microfluidic channel for flow rate and pressure measurements. The maximum flow rate and pressure values were obtained by processing the maximum value for each push and release. The beginning of the flow duration was set as a flow rate value that exceeded the background flow rate, and the end of the flow duration was set as a flow rate that met the background flow rate. The sensors were calibrated prior to use. The data were obtained and analyzed from three repetitive experiments.

### 2.6. Image of the Deflection of the Actuation Chamber

The images of the deflection of the actuation chamber under various timelines were obtained by a stereomicroscope (SZX16; Olympus, Tokyo, Japan) equipped with a charge-coupled device (CCD) camera (DP72; Olympus). The video was recorded under various pressing depths, and the images were captured from the recorded video according to the designated time.

## 3. Results and Discussion

### 3.1. System Setup of the Press Control Machine and Working Principle of PAMD

**Figure 1:**
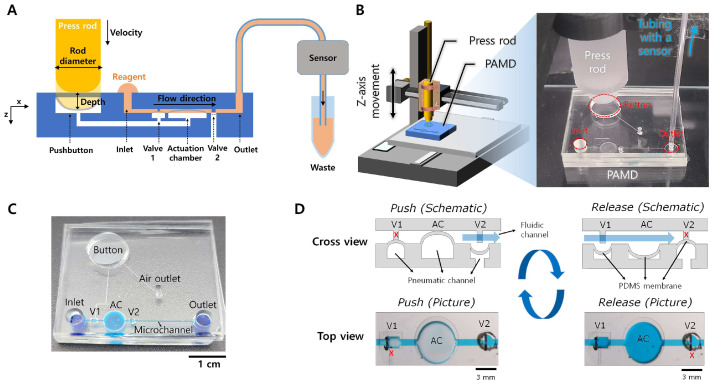
Schematic of the experimental setup and working principle of PAMD. (**A**) 2D schematic of PAMD connected with a sensor. The PAMD was activated via the customized press machine according to its designated parameters. (**B**) 3D schematic of the customized press control machine equipped with a press rod and a PAMD, and an actual image of the press rod and the PAMD. (**C**) An actual image of the PAMD filled with erioglaucine solution for visualization. The default length of the microchannel (L) was measured from V2 to outlet. (**D**) Actuation principle of the PDMS membrane in the PAMD.

The operation parameters of a customized press control machine, mainly pressing velocity and depth, were elaborately controlled to evaluate the flow rate profile in the PAMD. Figure 1A shows a cross-sectional view of the microfluidic device activated by the press rod connected to the customized pressing machine. The PAMD and the flow rate or pressure sensor were connected via a tube at the outlet. Figure 1B illustrates a schematic of the press control machine that allows elaborate control of x, y, and z-axis movement. The head of the machine was customized to be equipped with a 3D-printed rod to replace the pressing and releasing of the finger. The representative image of the PAMD is shown in Figure 1C. A simple straight microchannel was designed to evaluate the relationship between different parameters and the flow rate of the microchannel. The length of the microchannel was increased based on the straight microchannel length (L) to investigate how the channel dimensions affect the flow rate. Erioglaucine solution was injected into the microchannel for visualization. Figure 1D illustrates the working principle of the PAMD. When the button is pushed, the air is compressed inside the pneumatic channel, which deflects the PDMS membrane of valve 1 (V1) and the actuation chamber. Upon pushing the button, the pressure inside the pneumatic channel increases, which closes valve 1 (V1) and opens valve 2 (V2), resulting in a fluidic discharge from the actuation chamber to the outlet. In contrast, when the button is released, the air inside the pneumatic channel is decompressed, and the PDMS membrane of V1 and actuation chamber is expanded. At the same time, V2 is closed due to the decreased pressure in the fluidic channel, resulting in a fluidic charge from the inlet to the actuation chamber.

### 3.2. Validation of the Customized Press Control Machine

**Figure 2:**
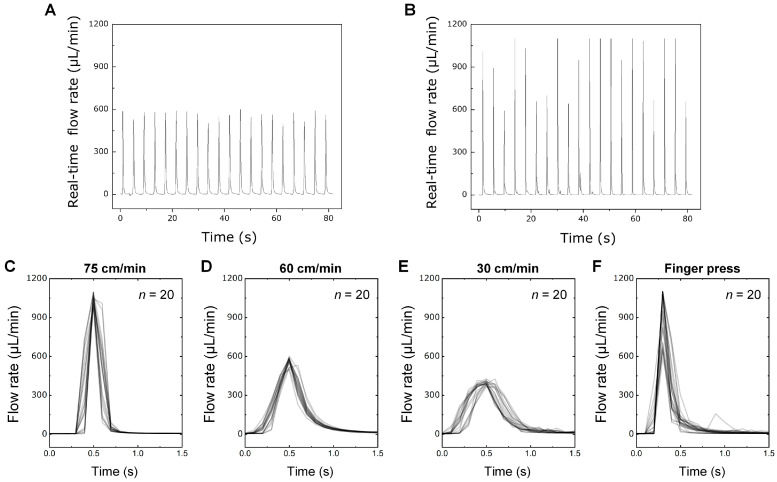
Evaluation of the customized press control machine and its performance comparison with human finger press. (**A**) Reproducibility of a flow rate using a customized press machine and (**B**) human finger press. Overlapped flow profiles of the PAMD using the press machine under the pressing velocity of (**C**) 75, (**D**) 60, (**E**) 30 cm/min, and (**F**) those using human finger press.

**Figure 3:**
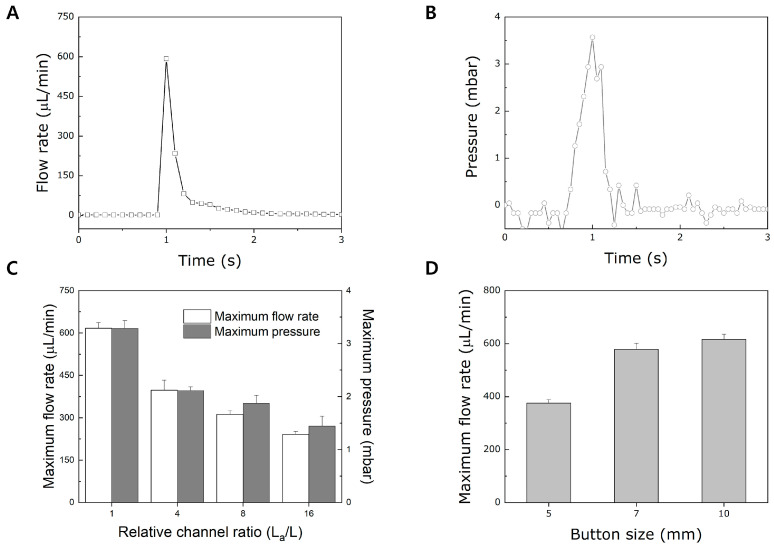
Evaluation of the flow behavior based on the design parameters of the PAMD. (**A**) A general flow rate and (**B**) pressure profile upon a single push and a release. (**C**) A correlation of microchannel length with maximum flow rate and pressure. (**D**) A relationship between maximum flow rate and button size.

To analyze the flow characteristics in the PAMD, we first evaluated the performance of the customized press control machine and then compared the fluid behaviors activated by the machine and human finger. Validation of the reproducibility of the flow rate of the press control machine was tested by generating 20 consecutive pushes and releases of a pushbutton. Figure 2A represents a real-time flow rate by the press control machine under the same condition that exhibits a stable flow profile and a reproducible maximum flow rate for each push and release due to sophisticated control of the pressing movement. On the other hand, the human finger-activated flow showed fluctuating maximum flow rates for every push and release due to difficulty in controlling the pressing velocity (Figure 3B). The maximum flow rates of the machine and finger groups were 560.01 ± 30.43 μL/h and 897.81 ± 190.08 μL/h, respectively. While the maximum flow rates of the human finger press remain unstable, those of the machine are relatively more stable, confirming their stability under precise parameters. Consequently, the customized machine was able to reduce the variation of the fluid behavior by the stable and reproducible activation of the button following the push and release command.

This validation was further investigated by changing the pressing velocities of the press control machine and overlapping each push and release peak. The overlapped flow profiles under pressing velocities of 75, 60, and 30 cm/min are shown in Figure 2C–E, respectively. The overlapped flow rates demonstrate significantly similar flow profiles and peak values under the same pressing velocity, demonstrating high reproducibility. The coefficients of variation (CoV) of the maximum flow rate under the pressing velocities of 75, 60, and 30 cm/min were 3.91%, 5.19%, and 4.09%, respectively. On the contrary, the human finger press shows an irregular maximum flow rate for each push and release (Figure 2F). When each flow profile was overlapped, a wide range of the flow rate peak was observable, and the CoV was 21.17%. Such data confirm the capability of the press control machine to produce a consistent flow behavior under the same conditions, validating its precise control of the PAMD for further evaluation.

### 3.3. Evaluation of Flow Behavior Based on the Design Parameters of PAMD

A general push and release of a pushbutton is shown in Figure 3A. When the button is pressed, a sudden increase in flow rate is noticeable due to the rapid deformation of the PDMS membrane of the actuation chamber by air compression in the pneumatic channel. As the flow rate reaches its maximum value, it dramatically decreases as there is no more liquid to dispense in the actuation chamber of the PAMD. We observed that such flow behavior was caused by applying pressure to the fluid in the actuation chamber upon a single push and release, as shown in Figure 3B. The pressure suddenly increases upon the press of the button and drops sharply as no more fluidic motion occurs. The data indicated that the deformation of the PDMS membrane pressurized the actuation chamber and caused fluid to flow from the inlet to the outlet in the PAMD.

Since flow rate and pressure are highly relevant to a channel dimension in microfluidics, various channel lengths of the PAMD using the machine under the same pressing condition were evaluated. To evaluate the channel resistance, the length of a simple straight microchannel shown in Figure 1C was set as L. The channel length was increased by multiplying 4, 8, and 16 to obtain the values of L_a_. All tests were carried out at a pressing velocity of 60 cm/min. Figure 3C shows the maximum flow rate and pressure based on various channel lengths. As the channel length increases, the fluidic resistance also increases, resulting in a decrease in the maximum flow rate and pressure and vice versa. Such a result denotes that the channel dimension is a significant factor influencing the flow behavior in the PAMD. Therefore, optimal channel dimension is required during the PAMD design for specific performance. Furthermore, one of the characteristics of the PAMD is that the fluidic motion is activated by pushing and releasing the pushbutton, which plays an important role in fluidic behavior. As shown in Figure 3D, various sizes of the pushbutton were tested under the same pressing velocity of 60 cm/min to assess the relationship between the pushbutton size and the flow behavior. Interestingly, as the pushbutton size increases, the maximum flow rate also increases. Such a phenomenon can be attributed to the amount of air compression. As the size of the pushbutton increases, the air volume in the pushbutton increases. Since more air molecules are available inside the bigger pushbutton, the air compression is larger as the size of the pushbutton increases, resulting in faster compression of the PDMS membrane of the actuation chamber. In this sense, designing an optimal pushbutton size plays another critical role in producing the desired flow rate in the PAMD.

### 3.4. Evaluation of Flow Behavior Based on the Pressing Velocity and the Rod Diameter

**Figure 4:**
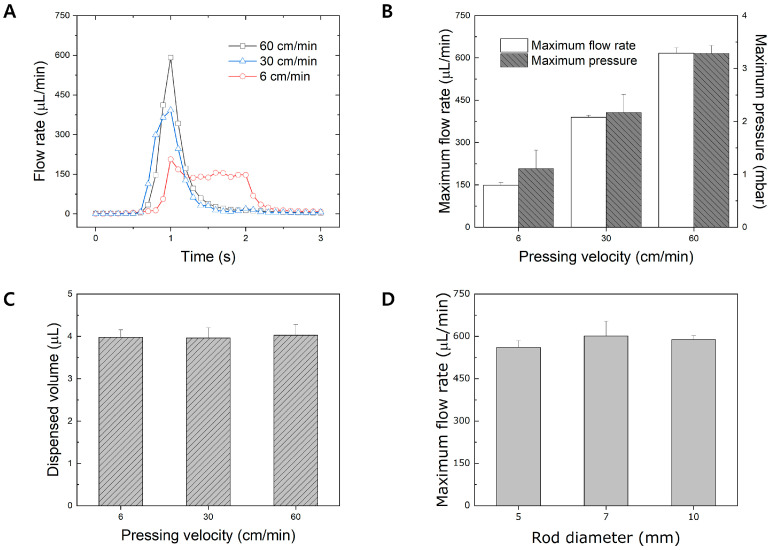
Evaluation of flow behavior based on pressing velocity and rod diameter. (**A**) A general flow rate profile based on various pressing velocities. (**B**) A correlation of pressing velocity with maximum flow rate and pressure. (**C**) A graph of the dispensed volume under various pressing velocities. (**D**) A graph of the average maximum flow rate versus rod diameter.

One of the variations between end-users when pressing and releasing a PAMD pushbutton is the pressing velocity. Three main pressing velocities (6, 30, and 60 cm/min) were selected via the customized press control machine to evaluate the relationship between pressing velocity and flow behavior. Figure 4A represents overlapped general flow rate profiles under the various pressing velocities. When the pressing velocity was set as 60 cm/min, a sharp increase and decrease in the flow rate was noticeable due to the fast compression of the PDMS membrane. When the pressing velocity was set as 30 cm/min, a sudden increase and decrease in the flow rate, not as much as 60 cm/min, was also observed. When the pressing velocity was set as 6 cm/min, the flow rate increased and a flow profile was maintained for 1 s before eventually decreasing. This phenomenon can be explained by a degree of compression and expansion of the PDMS membrane of the actuation chamber. As the pressing velocity increases, the fast compression of the PDMS membrane of the actuation chamber induces a sudden fluidic discharge, resulting in a sharp increase in the flow rate. Figure 4B demonstrates the relationship between pressing velocity and maximum flow rate. As the pressing velocity increases, the maximum flow rate also increases, and vice versa. A faster pressing velocity induces faster compression of the PDMS membrane of the actuation chamber, which embeds higher kinetic energy. Such high kinetic energy results in faster fluidic motion inside the microchannel. Likewise, the relationship between the pressure built inside the microchannel and the pressing velocity was also observed. Similarly, as the pressing velocity increases, the maximum pressure increases due to the higher kinetic energy built by the fast compression of the PDMS membrane. The results illustrate that pressing velocity is one of the main factors affecting the flow rate and profile. In this sense, an end-user may rudimentarily control the pressing velocity of a pushbutton in order to control the flow rate of the PAMD.

In addition, one of the advantages of the PAMD is the ability to control the desired fluid volume regardless of the end-users. The amount of dispensed volume is highly dependent on the volume of the actuation chamber. In this paper, we designed the actuation chamber to dispense 4 μL per single push and release. The dispensed volume upon the various pressing velocities was evaluated to confirm whether the pressing velocity influences the dispensed volume from a single push and release of a button. As shown in Figure 4C, the dispensed volume was consistent regardless of pressing velocity, demonstrating that pressing velocity does not influence the total dispensed volume. Such a result confirms that the dispensed volume is highly dependent on the degree of compression of the PDMS membrane of the actuation chamber, not on the pressing velocity. A finger size is another variation among end-users that may influence the flow behavior of the PAMD. Various diameters of the press rod were connected to the press control machine to mimic the various finger sizes among end-users. Figure 4D demonstrates the average maximum flow rate produced by the press control machine using various press rod diameters under pressing velocity at 60 cm/min. Despite the size variation in the rod diameter, the average maximum flow rate is consistent for every condition, demonstrating that the diameter of the rod does not influence the flow behavior of the PAMD.

### 3.5. Relationship of Pressing Depth and Flow Rate in the PAMD

**Figure 5:**
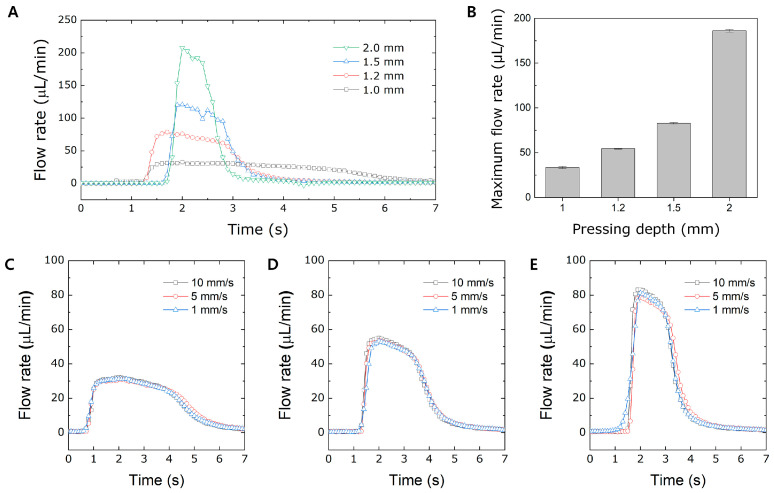
Investigation of the flow rate under various pressing depths. (**A**) A general flow rate profile based on various pressing depths. (**B**) A graph of the flow rate and pressing depth. The overlapped flow rate profiles by varying the pressing velocity while maintaining the pressing depth at (**C**) 1, (**D**) 1.2, and (**E**) 1.5 mm.

Since pressing depth also affects the degree of air compression of the pneumatic channel, the evaluation of the flow rate under various pressing depths was conducted using a press control machine. The pressing depth was elaborately controlled by the press control machine by setting the *z*-axis movement from 1.0 mm to 2.0 mm. When the pressing depth is deeper than 1.2 mm, the degree of air compression is sufficient, resulting in a sharp increase and decrease in the flow rate, as shown in Figure 5A. However, when the pressing depth becomes lower than 1.2 mm, the degree of compressed air also decreases, affecting the PDMS membrane deflection. When the degree of compressed air decreases, the rate of PDMS membrane deflection becomes slower and more consistent. The fluid flow continued for several seconds when the pressing depth was as low as 1.0 mm. In addition, the lower the pressing depth, the lower the maximum flow rate due to the slower deflection of the PDMS membrane of the actuation chamber. Under the same pressing velocity, the flow profile highly depends on the pressing depth.

The maximum flow rates were calculated based on various pressing depths under the same pressing velocity of 60 cm/min, as shown in Figure 5B. As the pressing depth increases, the maximum flow rate also increases due to increased air compression in the pneumatic channel of the PAMD. The pressing depth of the PAMD was further investigated by changing the pressing velocities under the same pressing depth (Figure 5C). The flow profile of each pressing velocity is almost the same at the same pressing depth, demonstrating that pressing velocity is negligible under the condition of a pressing depth lower than 1.5 mm. When the pressing depth is lower than 1.5 mm, it plays a dominant role in affecting the flow rate of the PAMD; however, when the pressing depth is higher than 1.5 mm, the flow rate is significantly affected by the pressing velocity, as shown in Figure 4B. Therefore, controlling the precise pressing depth is another important factor influencing the flow rate of the PAMD.

### 3.6. User-Friendly PAMD Using a Press Control Machine

**Figure 6:**
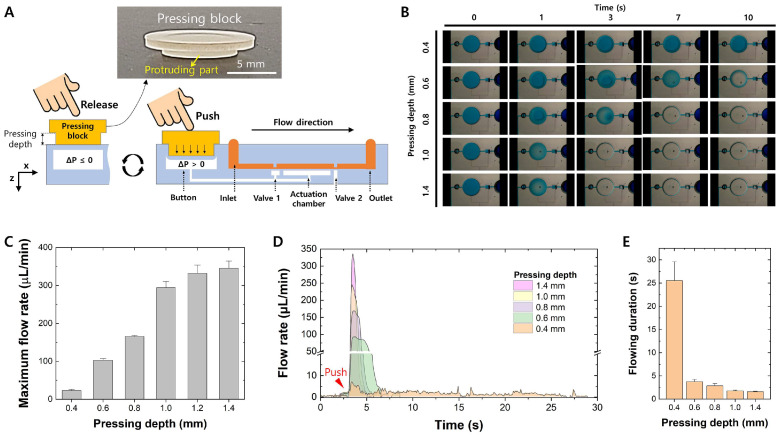
A user-friendly pressing block for sophisticated depth control and its evaluation. (**A**) A general schematic of using the pressing block to activate the PAMD. (**B**) Time-lapse images of the deflection of the actuation chamber based on various pressing depths. (**C**) A graph of maximum flow rate and pressing depth. (**D**) Representative flow rate profiles under various pressing depths using the pressing block. (**E**) Flow duration using various pressing depths of the pressing block.

Based on the evaluation of the phenomenon that precise control of pressing depth induces elaborate control of flow rate, a user-friendly, low-cost, and portable pressing block was developed to control flow behavior precisely, even by finger actuation (Figure 6A). The devised pressing block consisted of two parts: a main body and a protruding part. The dimensions of the main body stay constant while the height of the protruding part varies with an interval of 100 μm for precise control of pressing depth. The pressing block was designed to aid an end-user in easily controlling the flow rate of the PAMD. All the user needs to do is place the pressing block on the pushbutton of the microfluidic device and simply press and release the pressing block. Due to the height of the protruding part of the pressing block, the user can only press the pushbutton according to the designated pressing depth. In this sense, the pressing depth can be precisely controlled regardless of the end-users, allowing elaborate flow rate control. The devised pressing block was used to control the PDMS deflection based on various designated pressing depths. Figure 6B represents the time-lapse deflection of the PDMS membrane of the actuation chamber using the pressing block. The degree of compression varied depending on the pressing depth: the greater the pressing depth, the larger the deflection of the PDMS membrane, and vice versa. If the pressing depth was less than 0.6 mm, some liquid would be left in the actuation chamber due to partial compression of the PDMS membrane. When the pressing depth is as low as 0.4 mm, the PDMS membrane stayed almost immobile due to low air compression and showed a maximum flow rate of 24.621 ± 1.659 μL/min, as shown in Figure 6C. As the pressing depth increases, the PDMS membrane of the actuation chamber actively deflects, but the deflection rate varies based on the pressing depth. At the pressing depth of 0.6 mm, the full deflection of the PDMS membrane takes approximately 10 s due to the slow rate of air compression. However, as the pressing depth increases, the rate of full deflection of the PDMS membrane accelerates due to the increased rate of air compression. When the pressing depth exceeds 1.0 mm, the degree of PDMS membrane deflection becomes almost saturated. The minimum flow rate achieved while no liquid remains in the actuation chamber is approximately 160 μL/min when the pressing depth is equal to 0.8 mm. The maximum flow rate of the pressing block by a user under various pressing depths was also evaluated (Figure 6C). Unlike Figure 2B,F, which show an inconsistent maximum flow rate by the human finger press, the maximum flow rate stays stable with the aid of the pressing block due to the sophisticated control of press control. Representative flow profiles based on various depths using the pressing block are shown in Figure 6D. As pressing depth increases, the rate of air compression becomes faster, resulting in a sharper flow profile and a higher maximum flow rate. Interestingly, even at a pressing depth of 0.4 mm, a small amount of air was compressed in the pneumatic channel, inducing a flow of sub-microliter per minute in the microchannel for about 25 s. The flow duration was quantified based on various pressing depths with the aid of the pressing block, as demonstrated in Figure 6E. The flow continued until all the fluid inside the actuation chamber was dispensed upon the button press. When the pressing depth is as low as 0.4 mm, the deflection of the PDMS membrane is minimized, resulting in a continuous flow behavior until the built pressure is decreased. The lower the pressing depth, the longer the flow duration due to the slow rate of air compression. Due to the fast response of the PDMS membrane, the flow duration dramatically decreases as the pressing depth increases. The data indicate that the developed user-friendly and portable pressing block provided precise flow rate and profile control regardless of the end-users. The developed technique is advantageous compared to other fluid delivery methods, such as vacuum pumps or capillary channels, because vacuum pumps only work temporarily and capillary channels require additional surface treatment to maintain a low contact angle between the liquid and solid interface, which hinders various microfluidic applications. Moreover, both vacuum pumps and capillary channels suffer from a limited range of flow rates, making them unsuitable for wide microfluidic applications [36]. On the other hand, the developed precise fluid control technique merely requires a pressing block, needs no surface treatment, and can manage a variety of flow rates based on various pressing depths. Therefore, it can be further extended to the application of POCT, which requires elaborate flow control, such as inertial focusing [37,38] and nanoparticle synthesis [39,40,41].

## 4. Conclusions

The flow behavior of the PAMD was evaluated based on various parameters such as pushbutton size, channel geometry, pressing velocity, and pressing depth. The smaller the size of the pushbutton and the longer the channel length, the lower the maximum flow rate. Furthermore, the greater the pressing velocity and depth, the greater the maximum flow rate. Under the condition of pressing depth as low as 0.4 mm, the flow duration drastically increased due to the slow compression of the PDMS membrane of the actuation chamber. Based on the evaluation, we have developed a user-friendly and portable supporting tool that enables elaborate control of the PAMD by reducing user variation. The device performance was confirmed by demonstrating various flow behaviors with various heights of the protruding part.

## Data Availability

The data presented in this study are available on request from the corresponding author. The data are not publicly available due to privacy.
